# Science of 2.5 dimensional materials: paradigm shift of materials science toward future social innovation

**DOI:** 10.1080/14686996.2022.2062576

**Published:** 2022-05-06

**Authors:** Hiroki Ago, Susumu Okada, Yasumitsu Miyata, Kazunari Matsuda, Mikito Koshino, Kosei Ueno, Kosuke Nagashio

**Affiliations:** a Global Innovation Center, Kyushu University, Fukuoka, Japan; b Graduate School of Pure and Applied Sciences, University of Tsukuba, Ibaraki, Japan; c Department of Physics, Tokyo Metropolitan University, Hachioji, Japan; d Institute of Advanced Energy, Kyoto University, Kyoto, Japan; e Department of Physics, Osaka University, Osaka, Japan; f Department of Chemistry, Faculty of Science, Hokkaido University, Hokkaido, Japan; g Department of Materials Engineering, University of Tokyo, Tokyo, Japan

**Keywords:** 2.5 dimensional materials, 2D heterostructures, van der Waals interaction, moiré superlattice, interlayer nanospace, intercalation, bilayer graphene, transition metal dichalcogenide, hexagonal boron nitride, multidimensional materials

## Abstract

The past decades of materials science discoveries are the basis of our present society – from the foundation of semiconductor devices to the recent development of internet of things (IoT) technologies. These materials science developments have depended mainly on control of rigid chemical bonds, such as covalent and ionic bonds, in organic molecules and polymers, inorganic crystals and thin films. The recent discovery of graphene and other two-dimensional (2D) materials offers a novel approach to synthesizing materials by controlling their weak out-of-plane van der Waals (vdW) interactions. Artificial stacks of different types of 2D materials are a novel concept in materials synthesis, with the stacks not limited by rigid chemical bonds nor by lattice constants. This offers plenty of opportunities to explore new physics, chemistry, and engineering. An often-overlooked characteristic of vdW stacks is the well-defined 2D nanospace between the layers, which provides unique physical phenomena and a rich field for synthesis of novel materials. Applying the science of intercalation compounds to 2D materials provides new insights and expectations about the use of the vdW nanospace. We call this nascent field of science ‘2.5 dimensional (2.5D) materials,’ to acknowledge the important extra degree of freedom beyond 2D materials. 2.5D materials not only offer a new field of scientific research, but also contribute to the development of practical applications, and will lead to future social innovation. In this paper, we introduce the new scientific concept of this science of ‘2.5D materials’ and review recent research developments based on this new scientific concept.

## GRAPHICAL ABSTRACT

**Figure:**
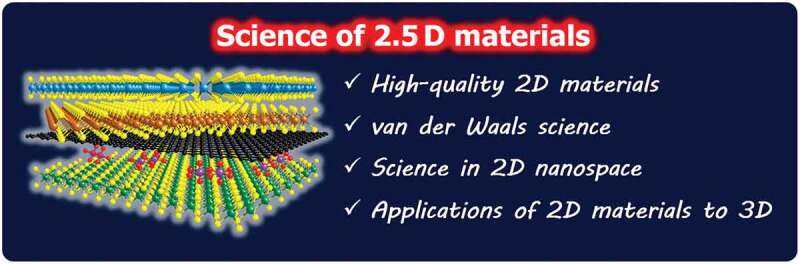

## 1. Introduction

Materials science is essential to our lives, because many advanced materials are used in, for example, electronics, automobiles, energy production and storage, healthcare, and information technologies. Graphene, a two-dimensional carbon sheet with single-atom thickness, was first prepared by Geim and Novoselov in 2004 using the so-called ‘Scotch tape method’ [1]. Monolayer graphene has attracted great interest from many researchers because it shows unusual physical phenomena, such as ultrahigh carrier mobility, the quantum Hall effect, and massless Dirac fermions [2]. In addition, many applications based on graphene have been proposed including flexible touch panels, integrated circuits, high-frequency transistors, sensors (chemical, biochemical, optical, and magnetic), gas barrier films, templates, and filter membranes – taking advantages of graphene’s high electrical conductivity and high carrier mobility, optical transparency, mechanical strength, and chemical stability [3].

**Figure 1.**
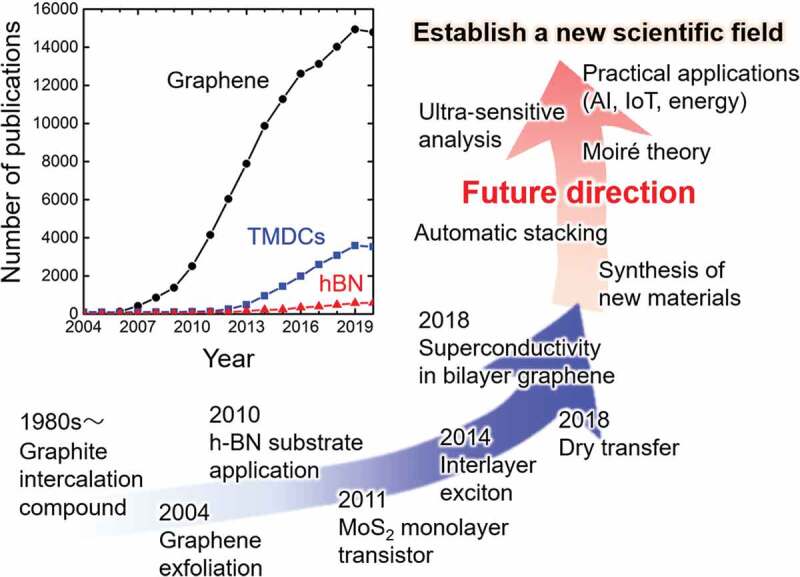
Trends and future directions of 2D materials research. Inset shows the number of publications about graphene, TMDCs, and hBN (the number of scientific papers whose titles contain each word was counted based on the ISI Web of Science database).

The discovery of graphene opened up a new research field — 2D materials. There are a number of 2D materials with different compositions: transition metal dichalcogenides (TMDCs), hexagonal boron nitride (hBN), and monoelement atomic sheets including silicene (Si), germanene (Ge), stanene (Sn), and black phosphorous (P) [4,5]. Theoretically, more than 1800 types of 2D crystals are predicted [6]. These 2D materials show unique physical properties that strongly depend on their chemical compositions and the number of layers. For example, molybdenum disulfide (MoS_2_) has an indirect band gap in the bulk crystal, but the monolayer form shows a direct bandgap in the visible range. Monolayer MoS_2_ also exhibits valley freedom and piezoelectricity. Figure 1 summarizes the research trends of 2D materials and the expected future research directions. Many exciting findings and achievements based on 2D materials have been reported. Therefore, as shown in the inset of Figure 1, the number of scientific publications has increased with time, reflecting this increased interest in 2D materials.

**Figure 2.**
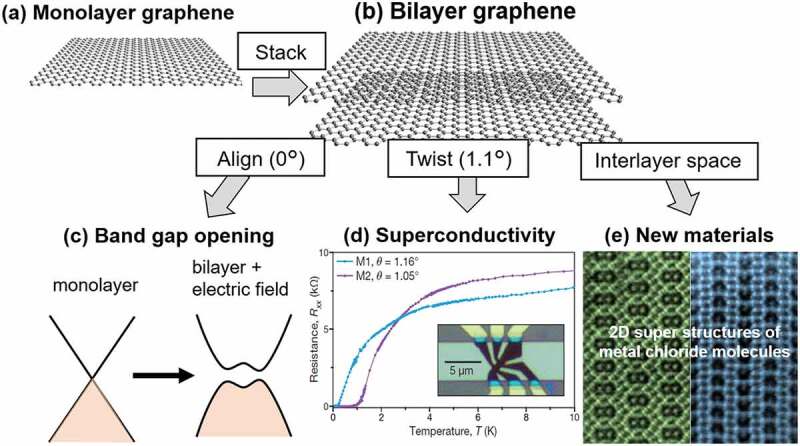
Schematic showing the scientific importance of stacking 2D materials. (a) monolayer graphene, (b) BLG. When stacked with the same angle, *i.e*. AB-stacking, the BLG shows band gap opening in the presence of vertical electric field, which is useful for semiconductor applications. (d) When the BLG is twisted with a magic angle (~1.1°), it becomes superconducting at low temperature. Reproduced with permission from Springer Nature [10]. (e) in the interlayer 2D nanospace of the BLG, new structures or new materials can be obtained. Reproduced with permission from Wiley-VCH^11^.

More importantly, now we are able to stack different 2D materials artificially by using advanced transfer techniques [7,8]. This means that we can control weak van der Waals (vdW) interactions so that the materials that can be stacked are no longer limited by lattice constant or composition. In addition, we can control the stacking angle once we know the orientation of the 2D crystals. These techniques essentially provide a new method to synthesize 2D crystals, thus offering new opportunities for breakthroughs in materials science. Some examples of stacking of two graphene sheets are presented in Figure 2. While monolayer graphene has no band gap, AB-stacked bilayer graphene (BLG) shows a band gap in the presence of a vertical electric field, making it an interesting material for application in semiconductor devices [9]. In contrast, taking advantage of the ability to change the stacking angle, twisted BLG (TBG) with a stacking angle of 1.1° shows a superconducting state at ⁓1 K which originates in the formation of a flat band owing to the long periodicity of the moiré superlattice [10]. The interlayer 2D nanospace, which is sandwiched by π-electrons of graphene, also provides an interesting platform for new materials science. We recently found that unique 2D superstructures of AlCl_3_ molecules, which are completely different from the structure of a bulk AlCl_3_ crystal, appear in the BLG interlayer space [11]. Exotic physical properties, such as interlayer excitons, interlayer *p*-n junction, interlayer tunnelling, and moiré excitons, have been demonstrated using stacks of 2D materials [12–15].

**Figure 3.**
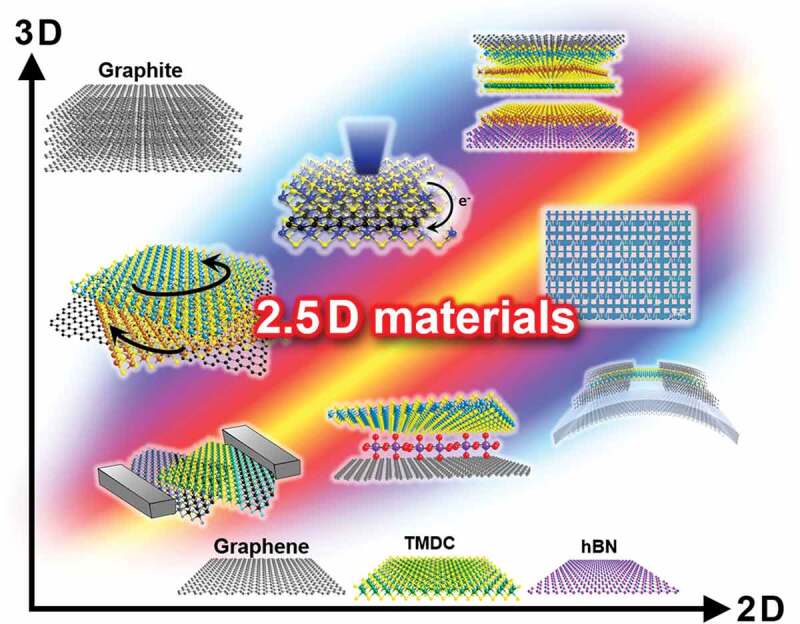
Our new concept of ‘2.5D materials.’ There is a large space to develop materials science between conventional 3D bulk materials and layered 2D materials.

We believe that the advancement of such integrated 2D materials will strongly impact materials science. As illustrated in Figure 3, there are symbiotic possibilities in the space between bulk 3D materials and 2D materials. We call these new materials and architectures ‘2.5D materials,’ where the 0.5D expresses the new dimension created by combining 2D materials through artificial manipulations, such as stacking, twisting, and connecting as well as through the 2D nanospace within 2D material stacks.

The term ‘2.5D materials’ also covers the integration of 2D materials with other dimensional materials – molecules and ions (zero-dimensional (0D)), nanotubes and nanowires (one-dimensional (1D)), and bulk crystals (three-dimensional (3D)). New research based on our 2.5D materials is expected to bring about a paradigm shift in materials science. The term ‘2.5D materials’ also means applications of 2D materials to our daily life, which is 3D. We can expect advances in 2.5D materials research to impact on many fields including IoT, artificial intelligence (AI), quantum computing, and energy production/storage/efficiency, thus contributing to our society.

Unlike traditional materials science, in which materials are synthesized based on rigid chemical bonds, in 2.5D materials the weak vdW interaction contributes to form solid materials. While self-assembly also provides materials based on vdW interactions, it generally gives the energetically most stable structure. The main difference is that our approach can control the stacking angle and stacked materials artificially in a highly controlled manner. Synthesizing new layered bulk materials via stacking massive numbers of 2D materials (such as 1,000–10,000 sheets) is challenging but is important to explore a new field of materials science. Inserting different 2D materials periodically in such bulk, layered materials is expected to greatly modify their electrical and optical properties. For this purpose, developing an artificial and automated stacking technology for large-area 2D materials is highly required. Although the transfer technique has been developed recently [8], the instruments are limited to small-scale stacking systems. In addition, the method to transfer a number of 2D materials without wrinkles and bubbles needs to be developed in order to clean interface for effective van der Waals interlayer coupling.

**Figure 4.**
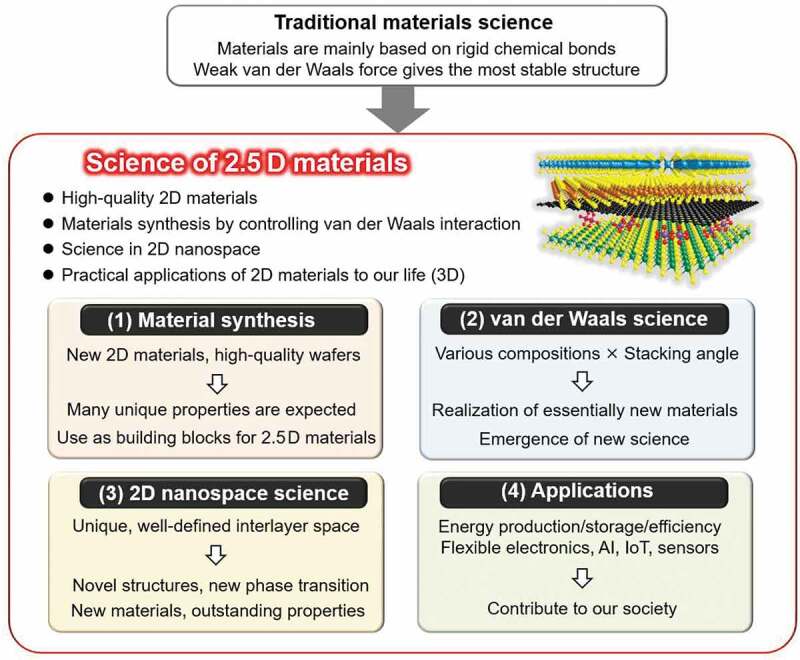
Details of science on ‘2.5D materials.’ The research topics and purposes are displayed. The difference between the traditional materials science research and the proposed 2.5D materials research is also explained.

Figure 4 shows an overview of the science of 2.5D materials, which includes four important research topics:

1. Material synthesis: making new 2D materials as building blocks of various 2.5D materials. In addition, production of high-quality, large 2D material wafers, such as hBN sheets, is also important.
2. van der Waals science: both vertical stacking and in-plane connection of 2D materials. In the former, combining different types of 2D materials with controlled stacking angles is expected to offer new phenomena, such as moiré physics, to establish a new scientific field. As there are infinite combinations of materials, theories that can predict the physical properties of new 2.5D materials are needed.
3. 2D nanospace: intercalation of molecules and ions to alter the physical properties of the host 2D materials, thus obtaining new ordered structures that could offer novel physical properties and phenomena. Moreover, 2D nanospace offers a unique reaction field for the growth of novel materials that cannot be obtained in usual environments.
4. Applications: Solar cells, batteries, flexible devices, quantum devices, and devices with very low energy consumption are expected.

In this article, we review recent scientific studies based on our newly proposed 2.5D materials science and present our perspective on the future of this exciting aspect of materials science.

## 2. Synthesis and chemistry of 2.5D materials

The growth and transfer of individual 2D materials are key processes for the fabrication of 2.5D materials. Among the various growth methods, chemical vapor deposition (CVD) is widely used to prepare large-area single crystals of 2D materials as well as their polycrystalline films. This will be focused on in Section 2.1. The CVD methods allow the synthesis of various 2D materials on substrates, and advanced CVD techniques can also grow in-plane and stacked 2.5D materials. However, stacking of 2D materials to prepare 2.5D materials has been more widely studied due to its simplicity. The state-of-art stacking methods are also discussed. This is followed by an overview of theoretical approaches to the design of various 2.5D materials, with an emphasis on predicting the effects of intercalation on materials properties (Section 2.2). Methods to intercalate molecules and ions in the interlayer nanospace as well as physical properties and applications of these hybrid systems are presented in Section 2.3.

### 2.1. CVD growth and assembly for 2.5D materials

This section reviews the studies on the CVD growth and transfer techniques used for typical 2D materials including graphene, hBN, and TMDCs. CVD process has been used to fabricate both vertical (stacked) and in-plane heterostructures of these 2D materials. In addition, the transfer technique enables the creation of vertical heterostructures, including twisted bilayers.

**Figure 5.**
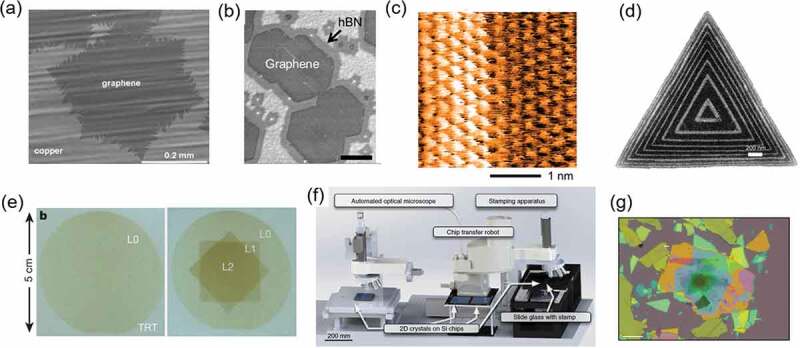
Growth and assembly of 2D materials. SEM images of (a) single-crystal monolayer graphene grown on Cu foil and (b) in-plane heterostructure of monolayer graphene and hBN grown on Cu foil. Reproduced with permission from American Chemical Society [18] and IOP Publishing [40]. (c) STM image of monolayer MoS_2_/WS_2_ in-plane heterostructure. Reproduced with permission from American Chemical Society [44]. (d) SEM image of monolayer WS_2_-WSe_2_ in-plane superlattice. Reproduced with permission from AAAS [46]. (e) Optical microscope images of wafer-scale monolayer (left) and a three-layer (right) MoS_2_ films obtained by the layer-by-layer transfer process. Reproduced with permission from Springer Nature [55]. (f) Robotic transfer system consisting of an optical microscope, stamping apparatus, and chip transfer system. (g) 29 alternating layers of graphene/hBN vertical vdW superlattice obtained by the robotic system shown in (f). Reproduced with permission from Springer Nature [8]. Scale bars are (a) 0.2 mm, (b) 5 μm, (c) 1 nm, (d) 200 nm, and (g) 20 μm.

In the case of graphene, CVD growth has mainly been conducted on metal surfaces that effectively decompose hydrocarbons, such as methane, through surface catalytic reactions [16,17]. In addition to the surface reactivity, the carbon solubility of metals is a major factor in controlling the number of graphene layers. For example, Cu foil is frequently used to grow monolayer graphene because of its very low carbon solubility [17], which facilitates the growth of large single crystals of monolayer graphene (Figure 5(a)) [18]. Recently, large-areas, fold-free films of single-crystal monolayer graphene were obtained by using Cu-Ni foil [19]. By increasing the Ni concentration in the Cu-Ni alloy, bilayer graphene, which can be regarded as the simplest form of 2.5D materials, has been also synthesized as a result of the high carbon solubility in the alloy and the suppression of the self-limiting property [20–22]. Similar CVD growth on metal surfaces has been also conducted for monolayer and multilayer hBN [23,24]. In these studies borazine and ammonia borane are widely used as the precursor of hBN. Highly-uniform multilayer hBN was achieved on a Ni–Fe alloy film, which greatly improved the optical property of monolayer WS_2_ grown on the hBN surface, when compared with that grown on a SiO_2_ substrate [25]. A 10 × 10 cm^2^ single-crystal monolayer hBN was reported to epitaxially grow on a Cu (110) vicinal surface [26]. CVD-grown graphene and hBN can be transferred from metal substrates to other substrates, such as silicon wafers, by the polymer-assisted transfer process [23,27].

Unlike graphene and hBN, highly-crystalline monolayer TMDCs can be directly synthesized on various non-metallic substrates, including SiO_2_ [28], sapphire [29], graphene/graphite [30,31], and hBN [32]. In early reports, monolayer TMDCs were frequently grown by CVD with transition metal oxides (such as MoO_3_ and WO_3_) and chalcogens (S, Se, and Te). These metal oxides usually require high temperatures ranging from 650–1100°C for the CVD process because of their low vapor pressures. More recently, metal and chalcogen precursors with high vapor pressures were also adopted to produce wafer-scale, uniform polycrystalline films of TMDC monolayers at lower growth temperatures by the metal-organic CVD (MOCVD) method [33]. Wafer-scale single crystals of TMDCs have been recently synthesized by using single-crystalline substrates [34] or seed crystals [35]. In addition to such surface growth, CVD growth of TMDCs occurs at the vdW interface between Au and SiO_2_ substrate surface, because the metal and chalcogen precursors can diffuse even at their interface [36].

In-plane heterostructures of 2D materials are one of the interesting groups of 2.5D materials. Such in-plane heterostructures were first demonstrated for monolayer graphene and hBN, where hBN grows from the edge of monolayer graphene by CVD (Figure 5(b)) [37–40]. In this process, boron-carbon bonds are preferentially formed at the lateral graphene-hBN interface, resulting in orientation-selective hBN growth from the graphene edge [40]. A similar growth process has been developed to produce in-plane heterostructures consisting of monolayer TMDCs, such as MoS_2_, WS_2_, MoSe_2_, and WSe_2_ [41–43]. A scanning tunnelling microscopy and scanning tunneling spectroscopy (STM/STS) study revealed an interface with atomically-sharp zigzag edges and a steep band offset around the interface in a MoS_2_-WS_2_ in-plane heterostructure (Figure 5(c)) [44]. Due to their composition-dependent band alignment [45], such semiconducting TMDC heterojunctions are particularly promising for future applications in electronics. Furthermore, the multistep and controlled growth of different TMDCs also enables the formation of ultranarrow nanoribbons and lateral superlattices (Figure 5(d)) [44,46].

Like in-plane heterostructures, vertical heterostructures can be also obtained by CVD. In 1984, Koma et al. reported that layered NbSe_2_ can be epitaxially grown on a MoS_2_ substrate, despite the large lattice mismatch of about 20%, introducing the concept of vdW epitaxy [47]. The vdW epitaxy has been observed in the CVD growth of various 2D materials, including graphene on hBN [48], and TMDC on graphene [31] (and on graphite [30], hBN [32], and TMDC [43]). Apart from vdW epitaxy, twisted bilayers are also frequently formed via the overlap of two monolayers with different orientations during growth, which has been characterized with electron and optical microscopies for graphene [49] and MoS_2_ [50]. It is noted that the growth of vertical heterostructures allows the formation of clean, atomically-flat vdW interfaces without any contaminations or wrinkle/bubble formation, overcoming some common problems in the transfer process described below. For example, CVD-grown TMDCs on graphite or hBN showed uniform photoluminescence (PL) with very narrow linewidths because of the suppression of unintentional lattice strain and carrier doping [30,32].

Nonetheless, the transfer process is the most used way to prepare vertical heterostructures by layer-by-layer stacking of 2D materials. In particular, hBN crystals have been widely used as insulating substrates with clean and atomically flat surfaces for other 2D materials [51]. Monolayer graphene on hBN shows higher carrier mobilities than it does on SiO_2_ substrates [52]. In recent years, the dry transfer process has been frequently used to stack multiple 2D materials to make, for example, a hBN/graphene/hBN vertical heterostructure [53]. Furthermore, to control the twist angle between two layers, the ‘tear and stack’ process of a single crystal of 2D material has been developed [54]. Wafer-scale vertical heterostructures with high uniformity were obtained by the layer-by-layer transfer of polycrystalline TMDC films grown by CVD (Figure 5(e)), in which bubble and wrinkle formation were suppressed by transferring in vacuum [55]. To create more complex vertical heterostructures, a robotic searching and assembly system was recently developed, as shown in Figure 5(f) [8]. This system allows the preparation of a vertical vdW superlattice consisting of 29 alternating layers of graphene and hBN (Figure 5(g)) [8]. Similar robotic assembly was also developed to produce an artificially-stacked material containing 80 layers of MoS_2_ made from the patterned CVD-grown MoS_2_ monolayers [56].

As shown above, the growth and transfer techniques have been advancing rapidly to fabricate various artificial structures based on 2D materials. In particular, the CVD process enables the syntheses of wafer-scale single crystals and superlattices of ultranarrow nanoribbons of 2D materials. The progress in transfer technique has also enabled moiré superlattices in twisted bilayers. A variety of 2.5D materials will be pioneered by the combination of these techniques and by applying them to other materials not only 2D materials.

### 2.2. Theoretical design of novel 2.5D materials

In this section, physical phenomena associated with the non-integer dimensionality (that is, the additional 0.5D) are discussed from the theoretical view point, together with theoretical predictions and design of novel 2.5D materials. Low-dimensional nanomaterials intrinsically possess hierarchical structures with themselves or other materials, where they act as elemental units, like atoms do in conventional solids [57–61]. Owing to the chemically-inert surfaces of the constituent nanomaterials, such as graphene and other 2D materials, large spacing between the constituent materials are inherent to such hierarchical structures, leading to an additional degree of freedom with which to tailor their physical properties. This additional degree of freedom endows such hierarchical materials with non-integer dimensionality. From the theoretical viewpoint, several pioneering works pointed out the importance of such non-integer dimensionality.

**Figure 6.**
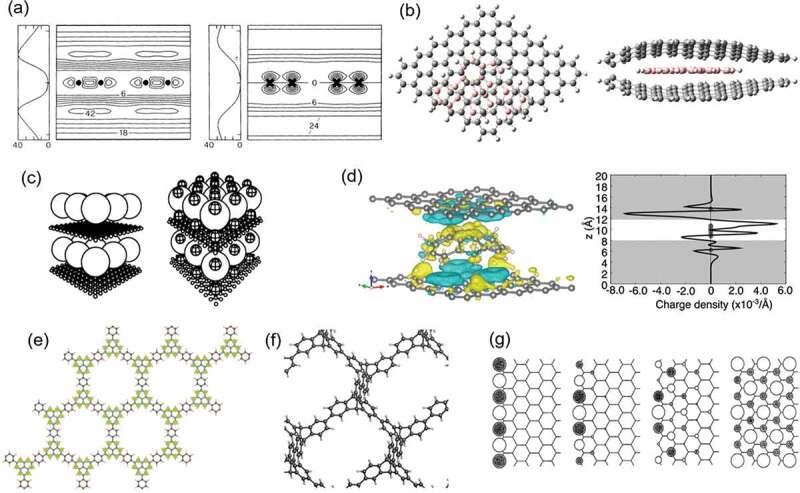
(a) Contour plots of the wave function of the lowest (left panel) and the second lowest (right panel) NFE states of an isolated graphene. The number shows the square of the wave function. Linear plots indicate the corresponding densities. Reproduced with permission from American Physical Society [62]. (b) an optimised structure of dibenzo-corannulene sandwiched by two graphene layers. Grey and pink balls denote C atoms belonging to graphene and dibenzo-corannulene, respectively, and white balls are H atoms. Reproduced with permission from American Chemical Society [63]. (c) Schematics of C_60_-graphene co-intercalation compound (left panel) and K-doped C_60_-graphene co-intercalation compound (right panel). Large, medium, and small balls denote C_60_ molecules, K atoms, and C atoms, respectively. Reproduced with permission from American Physical Society [64]. (d) Isosurfaces of electron-depleted (blue) and accumulated (yellow) regions of sumanene-intercalated bilayer graphene and the charge density distribution along z-axis. Reproduced with permission from American Chemical Society [67]. (e) Geometric structure and spin density of a copolymer of phenalenyl and phenyl groups. Reproduced with permission from Elsevier [68]. (f) Geometric structure of polymerized trypticene. Reproduced with permission from the Physical Society of Japan [69]. (g) Schematics of wave functions of the edge states at *k*=π, 8π/9, 7π/9, and 2π/3 (left to right). Reproduced with permission from IOP Publishing [70].

Graphene and other 2D materials are known to possess peculiar unoccupied electron states (nearly free electron (NFE) state or interlayer band); such states have their maxima in the vacuum space outside the 2D layered materials, as shown in Figure 6(a) [61–64]. Upon intercalating atomic layers of alkali or alkali-earth atoms between graphene layers, the NFE state shifts downward, owing to the attractive potential caused by metal ions, and crosses the Fermi level; this leads to superconductivity which would not have been expected from the simple sum of the electronic properties of the graphene layers and intercalated atomic layers [62].

Graphene and graphite can also form hybrid structures with molecules, such as fullerene (C_60_) and hydrocarbon molecules. In such hybrid structures, guest molecules are predicted to have 2D condensed forms which are rarely observed in their bulk phases. In addition, the confined nanoscale spacing between the graphene layers gives the novel molecular conformations that would be unstable under the ambient conditions. For example, as shown in Figure 6(b), a curved molecule, dibenzo-corannulene, becomes flat when enclosed in the two graphene layers [63]. A hybrid structure comprising C_60_ and graphite has been theoretically designed as a novel all-carbon compound with mixed dimensionality (Figure 6(c)) [64]. The electronic structures of the host graphene and guest C_60_ molecules are highly modulated when they are incorporated into these hybrid structures. Charge redistribution occurs by the substantial orbital hybridization between the NFE state of graphene and π states of C_60_. Another interesting possibility is sumanene, a bowl-shaped hydrocarbon molecule (C_21_H_12_) [65,66], which could form hybrid structures with graphene, offering unique electronic properties (Figure 6(d)). The electronic structure of the hybrid structures strongly depends on the sumanene conformation (i.e. whether it is ‘bowl’ or ‘flat’ [67]). When the sumanene molecule has the bowl shape between graphene layers, the dipole moment normal to the molecular plane of sumanene causes charge transfer, leading to electron and hole codoping in upper and lower graphene layers, respectively, with electron and hole densities of approximately 10^12^/cm^2^ (Figure 6(d)) [67].

Covalent networks of hydrocarbon molecules are a new type of 2.5D materials which have unique physical properties. Figure 6 show representative examples. As a result of the internal degree of freedom, a carbon-based network derived from phenalenyl and phenyl groups has a Dirac cone and Kagome bands at and above/below the Fermi level, respectively, owing to these constituent units and their arrangements (Figure 6(e)) [68]. Furthermore, two-dimensionally polymerized triptycene has Kagome bands at valence and conduction band edges, even though this covalent network contains sp^3^-hybridized carbon atoms (Figure 6(f)) [69].

Structural imperfections in covalent networks also play crucial roles in determining physical and optoelectronic properties of nanomaterials. An early theoretical calculation predicted that the graphene edges causes specific edge-localized electronic states (edge states) when the edge has a zigzag shape. This gives unique flat-band states at the Fermi level (Figure 6(g)) [70,71]. This result implies that the imperfections give an additional degree of freedom for the electronic properties of the resultant structures [68,72]. For example, in-plane heterostructures of graphene and hBN possess localized electronic states at the zigzag border between the two materials. In this case, the edge states of graphene and hBN hybridize each other at the zigzag border, resulting in the formation of bonding and antibonding edge states at their border [73,74].

### 2.3. Intercalation for the synthesis of 2.5D materials

**Figure 7.**
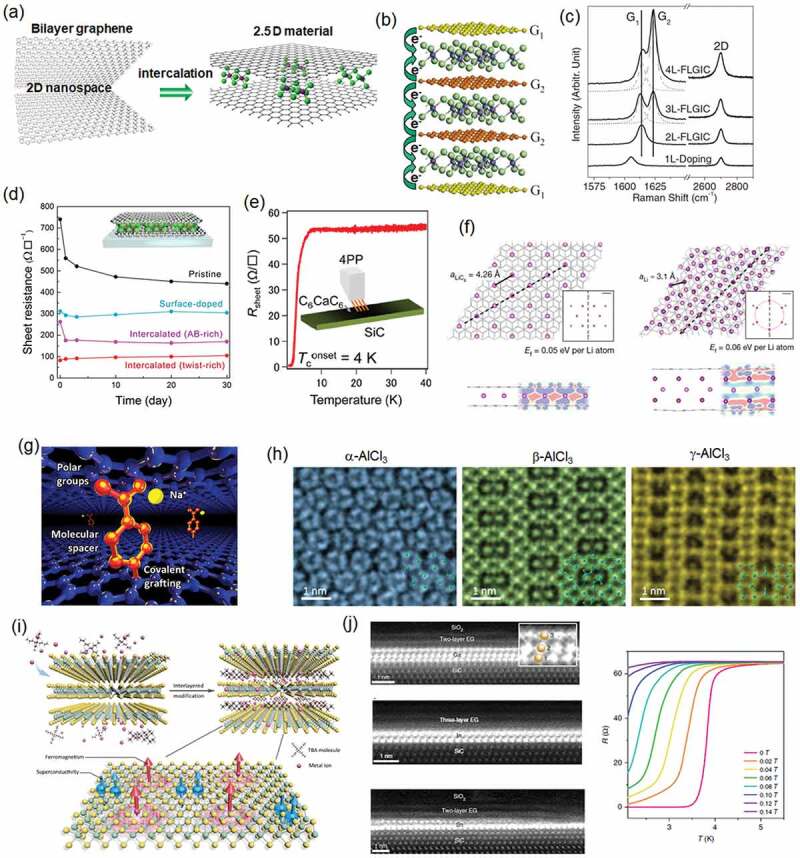
Intercalation in stacked 2D materials. (a) Concept of the intercalation within 2D nanospace. (b,c) Schematic and Raman spectra of monolayer, bilayer, and few-layer graphene intercalated with FeCl_3_ molecules. Reproduced with permission from Wiley-VCH [75]. There are two different graphene sheets with different doping levels; G_1_ and G_2_ indicate lower and higher doping levels, respectively. (d) Sheet resistance change of MoCl_5_-intercalated BLG stored in ambient condition. Reproduced with permission from Wiley-VCH (modified from ref. [79]). The BLG was grown by CVD method, and, by tuning the growth condition, AB- and twist-rich BLG were selectively synthesized. (e) Temperature dependence of the resistance of Ca-intercalated BLG grown on SiC. Reproduced with permission from American Chemical Society [81]. (f) Atomic models of Li ions stored in graphite (left) and BLG (right). Reproduced with permission from Springer Nature [83]. The simulation suggests higher density of Li in the BLG. (g) Illustration of Na intercalation in chemically modified graphene stacks. Reproduced with permission from AAAS [85]. Monolayer graphene sheets functionalized with 4-NBD molecules are stacked to form an electrode for the intercalation. (h) New AlCl_3_ structures observed inside the interlayer space of CVD-grown BLG. Reproduced with permission from Wiley-VCH [11]. (i) Intercalation of magnetic Co ions in the interlayer of TaS_2_ with the assistant of TBAC molecules. Reproduced with permission from Wiley-VCH [89]. (j) TEM images of atomic layers of Ga, In, Sn formed at the interface between graphene and SiC substrate. Right graph shows the magnetic field dependence of the superconductivity of the Ga layer. Reproduced with permission from Springer Nature [92].

Stacks of 2D materials provide unique well-defined 2D nanospace that is sandwiched by adjacent layers. As illustrated in Figure 7(a), this 2D nanospace can be used to intercalate molecules and ions, analogous to the extensively studied graphite-intercalation-compounds (GICs). In GICs, intercalation significantly alters the electrical, magnetic, and optical properties of the host graphite [57]. Similarly, the intercalation allows us to engineer the physical properties of host 2D materials. Thus, the intercalated 2D materials can be regard as ‘2.5D materials’.

The simplest 2D host material is bilayer graphene (BLG). FeCl_3_ molecules were intercalated into exfoliated BLG and few-layer graphene (FLG) in early studies [75–78], because FeCl_3_ is relatively easy to handle in ambient conditions owing to its stability. The intercalation of FeCl_3_ induces hole doping in the BLG and significantly reduces the sheet resistance of the host BLG. Compared with the widely used surface doping of graphene, higher stability is expected for intercalated materials, because the intercalants are protected by graphene layers. In the case of BLG and FLG, the intercalation can be monitored by the shift of the Raman G-band, which is sensitive to the carrier density. As shown in Figure 7(b), the G-band upshifts to higher wavenumbers with increasing carrier concentration [75]. The graphene sandwiched by FeCl_3_ layers (G_2_ in Figure 7(c)) shows a larger G-band shift than that of the graphene which only faces FeCl_3_ on one side (G_1_). The ability to evaluate the doping degree using Raman spectroscopy is a strong advantage of graphene – in other 2D materials it is not as easy to determine their doping levels.

In the previous research, most of the BLG and FLG was prepared by exfoliation of bulk graphite. This limits the size and stacking order of the resulting graphene; exfoliation gives small graphene flakes with mostly AB stacking order. Kinoshita et al. investigated the intercalation of MoCl_5_ molecules in large-area BLG sheet which was grown by CVD [79]. As shown in Figure 7(d), they discovered that TBG with high twist angles is more amenable to intercalation than BLG with AB stacking, resulting in lower sheet resistance of the intercalated TBG. This can be accounted for by the weaker interlayer coupling in large-angle TBG. Because the CVD growth gives a large BLG sheet, it was possible to fabricate large-area organic solar cells using intercalated BLG instead of an indium tin oxide (ITO) electrode [79]. The low sheet resistance of FLG obtained by MoCl_5_ intercalation enabled its use as interconnects to replace commercial Cu wires [80]. Because Ca-GIC shows the highest superconducting-transition temperature (*T*_c_) of 11.5 K among GICs, BLG was also used to intercalate Ca ions [81]. By using BLG grown on a SiC substrate, Ichionokura et al. prepared Ca-intercalated BLG, which showed a superconductor transition at 2–4 K (Figure 7(e)) [81]. Although the *T*_c_ is lower than that reported for Ca-GIC, there is a still chance to increase the *T*_c_ by using hBN substrate or other approaches. Graphene layers were also used as a template as well as a protecting layer for NbSe_2_ 2D superconductor [82].

Relevant to energy storage, Kühne fabricated an electrochemical monitoring setup and found superdense ordering of Li ions in BLG with a much higher Li density than that seen in Li-GIC (C_6_Li) (Figure 7(f)) [83]. The same group also reported that the diffusion rate of Li ions in BLG is much faster than that in graphite [84]. These interesting results open a new opportunity to produce high-performance rechargeable batteries based on 2.5D materials. It is known that graphite cannot host Na ions, even though Na-ion batteries are expected to be cheaper and to use more abundant resources than the current Li-ion batteries. Recently, by stacking CVD-graphene that had been functionalized with 4-nitrobenzene diazonium tetrafluoroborate (4-NBD) and reduced, the intercalation of Na ions has been realized [85]. Figure 7(g) depicts how the 2D nanospace that had been expanded by the 4-NBD molecule enables the intercalation of bulky Na ions, while attracting them to the -NH_2_ groups.

It is noted that 2D nanospace can offer a unique stage to explore materials science. One example can be found in the work by Y.-C. Lin et al. [11]. They intercalated AlCl_3_ molecules into CVD-BLG and observed their atomic structures using a scanning transmission electron microscope (STEM). Interestingly, they discovered three new AlCl_3_ crystalline structures in the interlayer 2D nanospace as shown in Figure 7(h), which are completely different from the structure of the bulk AlCl_3_ crystal [11,86]. In addition, these structures showed phase transitions upon excitation by an electron beam. The reason for these new crystalline structures is not clear, but it may be related to the surrounding π-electrons and/or a pressure effect suggested for the molecules confined between the graphene layers [87]. Experimental finding of such new structures demonstrates the potential of 2D nanospace and the strong impact on traditional GIC research. Graphene-based liquid cells that contain liquid, such as water, between graphene layers can be also regarded as 2.5D systems. Such cells enable *in situ* observation of liquid phase reactions inside a transmission electron microscope (TEM) [88].

The use of 2D nanospace is not limited to BLG and FLG. For example, Li et al. proposed ‘imprinting’ ferromagnetism by intercalating magnetic metals into layered TaS_2_ (Figure 7(i)) [89]. Given that tetrabutylammonium chloride (TBAC) can be intercalated into a TaS_2_ crystal, they facilitated the cointercalation of Co ions into TaS_2_ layers by dissolving CoCl_2_ in the TBAC solution. This allowed the experimental observation of the magnetic properties of the Co-intercalated TaS_2_. An electrochemical method has also been used to intercalate molecules into MoS_2_ layers [90]. Interestingly, the intercalation of cetyltrimethylammonium bromide (CTAB) induced the phase transition of MoS_2_ from the semiconducting 2 H phase to the semimetallic 1T phase; these are examples where both the intercalant and the host 2D material offer unique physical and chemical properties.

As well as the interlayer spaces within 2D materials, the nanospace between the 2D material and substrate surface is also an interesting place to intercalate molecules and solid materials. Hayashi et al. reported the synthesis of 2D Sn at the graphene-SiC interface [91]. Later, Briggs et al. demonstrated the formation of 2D Ga, In, or Sn layers at the interface by applying plasma treatment (which gave entry pores for these elements) prior to the evaporation [92]. Figure 7(j) shows cross-sectional TEM images of these 2D monoatomic layers. Moreover, the 2D Ga layer exhibited a superconducting transition, which showed different behavior from that of Ga bulk crystals, suggesting that the 2D Ga layer confined in 2D nanospace is unique.

## 3. Physical properties of 2.5D materials

Stacking of two layers of 2D materials brings out novel physical properties that cannot be expected from each of the constituents. This is because we have new degrees of freedom, such as combination of 2D materials, stacking (twist) angles, and valleys, which are obtained through the stacking of layered materials by the methods described in Section 2.1. In other words, the possibility of the control of van der Waals interaction makes 2.5D materials unique and exciting. In particular, twist angles or different lattice constants in stacked bilayers give moiré superlattices which open a new scientific field. In this section we focus on new physics brought by moiré superlattices in stacked bilayers, followed by the experimental observations of intriguing physical properties that have appeared in various types of 2.5D materials.

### 3.1. Moiré physics in 2.5D materials

Moiré superlattices are a novel type of nanoscale materials in which individual 2D materials are stacked on top of each other without lattice matching. A striking feature of this class of materials is that physical properties are strongly dependent on the moiré pattern caused by the interference of the lattice structures. The moiré period, typically a few to tens of nanometers, significantly modifies the band structure of the host materials, giving rise to a number of remarkable properties that cannot be observed without stacking. In the following, we review recent scientific studies on various moiré superlattices.

**Figure 8.**
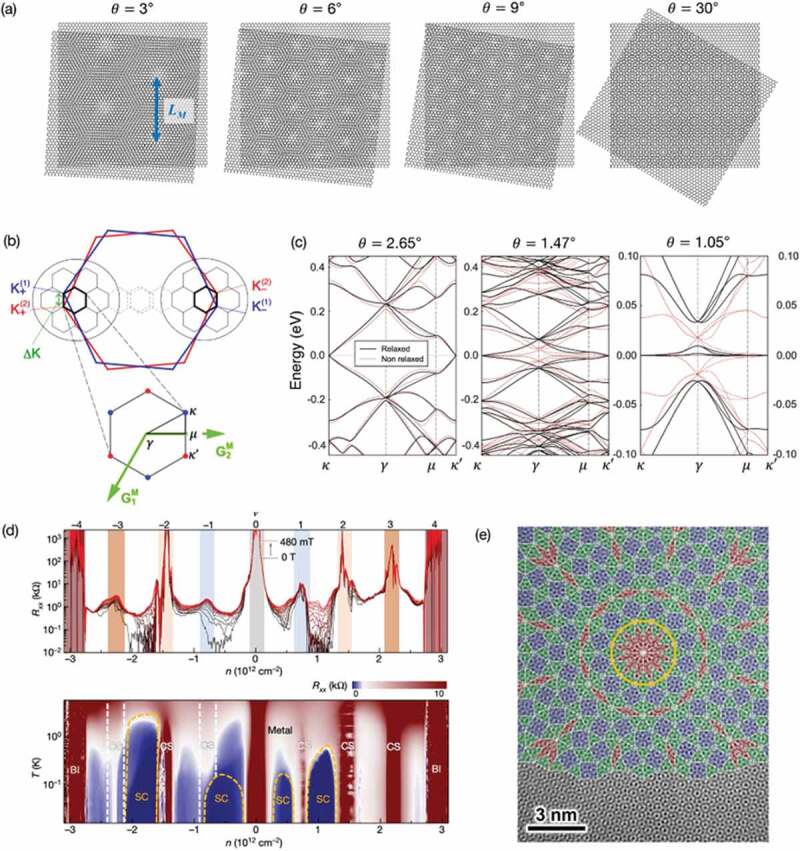
(a) Atomic structures of TBG with different twist angles. (b) Schematics of the Brillouin-zone (BZ) folding of TBG (10 degree), where red and blue hexagons represent the BZ of individual graphene layers, and small black hexagons are that of the moiré superlattice. (c) Band structures of low-angle TBG. Reproduced with permission from American Physical Society [98]. (d) (Top) Longitudinal resistance plotted against carrier density at different perpendicular magnetic fields from 0 T (black trace) to 480 mT (red trace). (Bottom) Color plot of longitudinal resistance measured against carrier density and temperature, showing different phases including metal, band insulator (BI), correlated state (CS) and superconducting state (SC). Reproduced with permission from Springer Nature [101]. (e) A false-colored TEM image of 30-degree TBG mapped with 12-fold Stampfli-inflation tiling. Reproduced with permission from AAAS [127].

A typical example of moiré superlattice is TBG [10,93–103], in which, as described earlier, a pair of graphene layers are rotationally stacked with an arbitrary twist angle (Figure 8(a)). The moiré period of TBG is inversely proportional to the twist angle, and is ⁓15 nm at 1 degree. In the electronic structure of TBG, the Dirac cones of individual graphene layers are strongly hybridized by the superlattice interlayer coupling (Figure 8(b)), and the electronic spectrum is rearranged into a series of moiré subbands (Figure 8(c)). Generally, the energy width of the moiré subbands decreases as the twist angle is reduced, and, at a so-called ‘magic angle’ near 1 degree, a dispersionless ‘flat band’ appears [94]. Remarkably, the magic angle TBG exhibits superconductivity when the flat band is partially filled (Figure 2 and 8(d)) [10,99–101]. The superconductivity is believed to be caused by the electron-electron interaction and the band flatness. In a flat band, generally, the kinetic energy is quenched, and hence the physical properties are dominated by the electron-electron interaction. Besides the superconductivity, magic angle TBG shows various phases depending on the electron density, including correlated insulating phases [10,99–101] and magnetic phases [102,103], which are all expected to originate from electron-electron interactions. The superconductivity and correlated phases have been also found in other moiré graphene systems, such as twisted double bilayer graphene (*i.e*. a twisted pair of AB-stacked bilayer graphene) [104–109], and twisted trilayer graphene [110–113], where the band flatness is assumed to be essential, as it is in TBG.

The moiré superlattice is not limited to graphene. For instance, a twisted stack of TMDCs also gives a moiré superlattice with unusual optical properties. A monolayer of TMDC (*e.g*. MoS_2_, MoSe_2_, WS_2_, WSe_2_) is a direct band gap semiconductor in which an optically excited electron-hole pair forms an exciton. When two monolayers of TMDC are rotationally stacked, an optical excitation gives rise to localized excitons trapped by the moiré pattern [12,114,115]. These trapped excitons could be applied to information storing devices.

Another well studied moiré superlattice is a composite system of graphene and hBN, where the slightly different lattice constants of graphene and hBN makes a 15 nm moiré pattern without rotation [116–118]. Here, the graphene’s Dirac cone is strongly modulated by an effective potential induced by the hBN, resulting in formation of minigaps in the Dirac cone [119,120–123]. When the Fermi energy locates in a minigap, the system exhibits the valley Hall effect [124]. This is the Hall effect analogue which occurs in the absence of magnetic field, where electrons of the K and K’ valleys of graphene move in opposite directions perpendicular to an applied electric field. This phenomenon is attributed to the emergent Berry curvature induced by the inversion-symmetry breaking potential from hBN. It was also reported that superconductivity is observed in ABC-stacked trilayer graphene on hBN, where flat bands are formed in ABC-graphene’s band by the hBN superlattice modulation [125].

Most of the 2D superlattices reported in the literature, including the examples presented above, are characterized by long-range moiré patterns (~ tens of nm) which are much longer than periods of atoms in crystals. Theoretically, such systems can be treated by an effective moiré theory [93–97], which extracts the moiré length period while coarse-graining the atomic-scale structures. However, when the twist angle is too large, or the atomic periods of the host materials are not close to each other, the long-range and short-range length scales cannot be separated. Then, the system becomes essentially quasiperiodic, in which incommensurate periodicities coexist, and the Bloch theory is not applicable [126]. A representative example of this situation is a 30-degree rotated twisted BLG, which can be fabricated by an epitaxial growth on a SiC surface [127]. As shown in Figure 8(e), the system has the striking feature of a 12-fold rotational symmetric quasicrystal, which is intimately related to quasiperiodic tiling in mathematics [127,128]. Also, quasiperiodic features generally emerge in trilayer systems, such as hBN/graphene/hBN [129–136], where two moiré patterns coexist.

In these non-periodic situations, theoretical description of physical properties is limited by the inapplicability of the Bloch theorem. There have been several theoretical attempts to describe the electronic structure of quasiperiodic 2D superlattices. For the 30-degree TBG mentioned above, the 12-fold rotational symmetric electronic structure can be successfully described by quasi-bands defined in quasi momentum space [128]. Quite recently, it was shown that energy gaps in 2D quasiperiodic systems can generally be characterized by a set of topological numbers called the second Chern numbers, which can be interpreted as quantized integers in the high-dimensional quantum Hall effect [137]. These concepts serve as a fundamental framework to describe the physical properties in quasiperiodic twisted 2D systems, where the Bloch theory is not applicable. The above-mentioned, highly controlled nanoscale architectures consisting of 2D materials, such as graphene, hBN, and TMDC, that exhibit extraordinarily properties can also be categorized as 2.5D materials.

### 3.2. Physical properties of 2.5D materials

**Figure 9.**
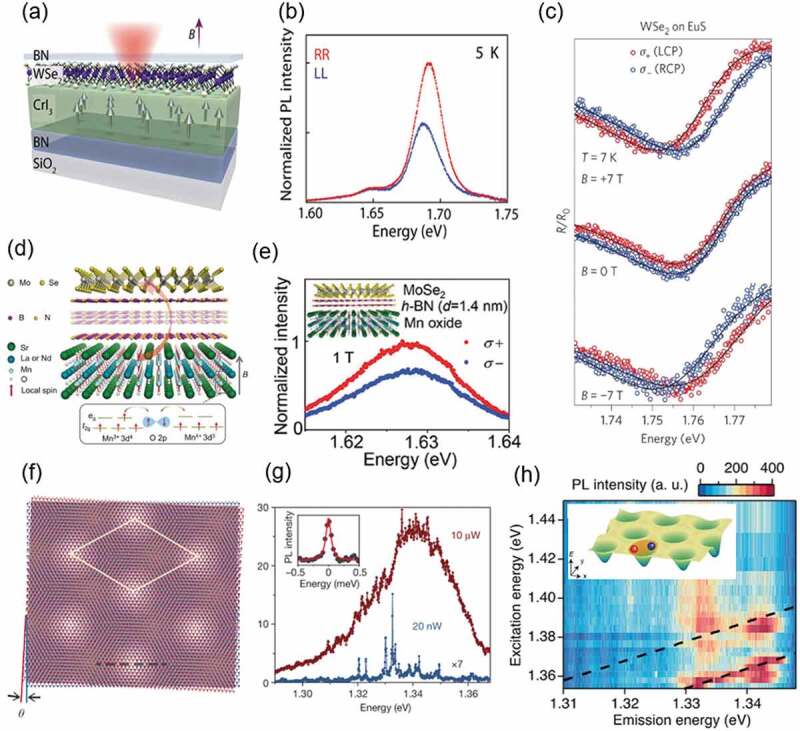
(a) Schematic of the artificial heterostructure of semiconducting monolayer (WSe_2_) and magnetic material (CrI_3_). Reproduced with permission from AAAS [143]. (b) Spectrum showing circularly-polarized PL from excitonic states in monolayer WSe_2_, which suggests valley-Zeeman splitting induced by a magnetic proximity effect from the ferromagnetic CrI_3_. Reproduced with permission from AAAS [143]. (c) Valley-Zeeman splitting induced by a magnetic proximity effect from ferromagnetic EuS. Reproduced with permission from Springer Nature [145]. (d) Schematic of vdW heterostructure of a semiconductor monolayer (MoSe_2_) and a perovskite transition metal oxide ((La._8_Nd._2_)_1.2_Sr_1.8_Mn_2_O_7_) with a hBN layer. Reproduced with permission from Wiley-VCH [146]. (e) Large valley-Zeeman splitting and polarization in monolayer MoSe_2_ on perovskite Mn oxide. Reproduced with permission from Wiley-VCH [146]. (f) Schematic of the atomic arrangement in the moiré superlattice formed in a heterobilayer with twist angle *θ*. Reproduced with permission from Springer Nature [114]. (g) Low temperature PL spectra of moiré superlattice in MoSe_2_/WSe_2_ heterobilayer, measured at low (blue: 20 nW) and high (dark red: 10 µw) optical power densities. Inset shows an expanded energy scale of a sharp PL spectral line, with linewidth ~100 μeV. Reproduced with permission from Springer Nature [114]. (h) 2D photoluminescence excitation (PLE) map. The two-resonance excess energies of 24 and 48 meV are indicated by the black dashed lines. Reproduced with permission from American Chemical Society [13].

The novel physical properties that emerge at the nanoscale interface between the atomically thin 2D materials in vdW heterostructures are an important research topic for both science and application of 2.5D materials. Artificial vdW heterostructures based on semiconducting 2D materials have been most widely studied because of their importance in electronics and photonics [138–140]. The valley degrees of freedom coupled with the spin (spin-valley locking) in the monolayer semiconductor enables selective circularly polarized excitations and emissions from optically generated excitonic states (excitons and trions) at the equivalent K (−K) valley without magnetic fields [141,142]. The interface between monolayer semiconducting and magnetic materials in the vdW heterostructures provides a novel platform for investigating optically excited states with spin-valley locking properties. Figure 9(a) shows a schematic of an artificial vdW heterostructure composed of a monolayer semiconductor (WSe_2_) and a ferromagnetic insulating material (CrI_3_) [143]. The vdW heterostructure (WSe_2_/CrI_3_) exhibits large energy splitting of excitonic states (valley-Zeeman splitting) and emission of circularly-polarized light (valley-spin polarization) due to breaking of valley degeneracy under the effective magnetic field [144] arising from local-spins in ferromagnetic CrI_3_ (Figure 9(b)). A similar result was also reported for a vdW heterostructure with a different ferromagnetic insulator, EuS (Figure 9(c)) [145]. The interaction of valley spin-polarized excitons (trions) and ferromagnetic spins can be further enhanced and controlled via proximity magnetic-exchange effects. The tunable valley-spin polarization and large valley-Zeeman splitting with the hBN thickness at the interfaces (*i.e*. spacer layer) have been demonstrated in the vdW heterostructure of a semiconductor monolayer (MoSe_2_) and a ferromagnetic, perovskite transition metal oxide ((La._8_Nd._2_)_1.2_Sr_1.8_Mn_2_O_7_), as shown in Figure 9(d,e) [146].

The universal phenomena of overlapping and tilting of two similar periodic patterns are frequently seen in our daily life, and are called moiré interference patterns, as described in the previous section. Moiré quantum interferences across a wide range from the atomic (or nano)- to macro-scale play an important role in science, such as in superconducting quantum interference devices [147], superconductivity in solids [10,100], and others [102]. The moiré physics in the interference of twist-stacked atomically thin 2D materials provides novel effects that are not seen in other materials; finding such effects is one of the important research targets in the science and engineering of 2.5D materials. The moiré pattern arising from the spatially varying atomic registry, as shown in Figure 9(f), causes periodic trap potential for excitons in vdW heterostructures made by twisted stacking of two semiconductor monolayers [12,114,115,148].

Figure 9(g) shows typical low-temperature PL spectra of a MoSe_2_/WSe_2_ heterobilayer, measured at low (blue: 20 nW) and high (red: 10 µW) optical power densities [114]. Several sharp lines are clearly observed in the PL spectrum under the excitation power density of 20 nW. The inset shows a single sharp (linewidth ~100 μeV) PL spectral line on an expanded energy scale. The extremely sharp and randomly distributed PL peaks in the spectra stem from recombination of excitons trapped in a moiré potential (moiré excitons), which are very different to the broader emission peak from 2D excitons in the spectrum of monolayer WSe_2_ (MoSe_2_). The precise energies of excitons trapped in the moiré potential well, which are sensitively affected by the small differences in local environment in each potential well, have a Gaussian distribution. A Gaussian broadened peak, with multiple smaller sharp peaks, was observed in the PL spectrum under the high optical power density (10 µW), because each moiré potential well is occupied by excitons.

The PL excitation (PLE) spectra provide complementary information about the optical absorption of moiré excitonic systems. Figure 9(h) shows the 2D PLE spectral map of a MoSe_2_/WSe_2_ heterobilayer, collected from the resonant PL spectra as a function of excitation photon energies [13]. The PLE map suggests that the PL spectrum change strongly depends on the excitation energy, and that the optical absorption occurs under near-resonant conditions. Significant PLE signals were observed near the tilted dashed lines with excess energies of ~ 24 and ~48 meV, where the excess energy is defined by the difference between emission and excitation photon energy. The energy difference of 24 meV observed in the PLE resonance is close to the energy of the phonon modes in MoSe_2_ and WSe_2_, suggesting that the moiré excitons resonantly couple with their phonons.

The precise probing techniques that have been developed to examine the nanoscale interface and moiré excitonic systems formed in novel vdW heterostructures will also contribute to the emergence of new physical properties and further understanding of the physics of 2.5D materials.

## 4. Applications of 2.5D materials

In the previous chapter, we discussed the science of 2.5D materials, mainly focusing on their synthesis and physical properties. Integration of various 2D materials with controlled angles, stacking of a large number of 2D materials (*e.g*. >100 layers with controlled compositions), intercalation in the interlayer nanospace, 3D macrostructures made of 2D layered materials – all these can be considered as 2.5D materials – have enormous potential for applications, such as quantum information, electronics, automobiles, energy, biotechnology, and environmental protection. In addition, most of the applications of 2D materials require solid substrates (3D materials) in which interface engineering is essential to bring out the intrinsic physical properties of the 2D materials. Therefore, mixing or integration of materials across dimensions is also crucial for the future development of potential applications. In this section we focus on electronic, energy, and photonic applications of 2.5D materials, all of these take advantage of their unique geometrical structures and physical properties.

### 4.1. Electronic applications of 2.5D materials

**Figure 10.**
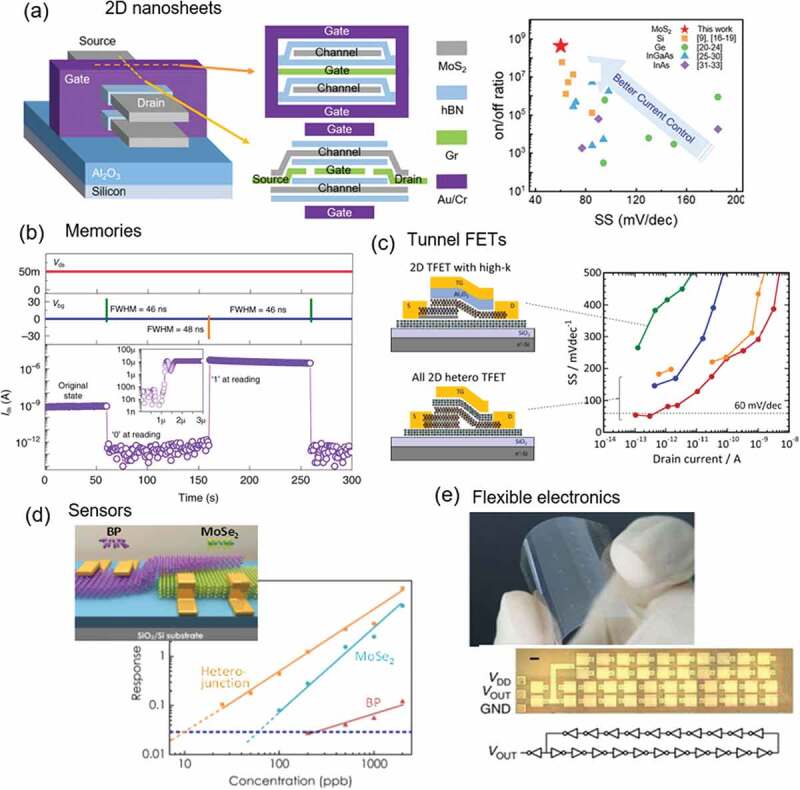
(a) Schematic 2D nanosheet heterostructure (left) and current on/off ratio as a function of SS (right). Reproduced with permission from Wiley-VCH [152], the references referred in the right panel can be found in this reference). (b) Ultrafast program/erase operation in MoS_2_/hBN/multilayer graphene heterostructure NVM. Reproduced with permission from Springer Nature [160]. (c) SS lower than thermodynamic limitation of 60 mV/dec achieved in *n*-MoS_2_/*p*^+^-MoS_2_ TFET heterostructured with hBN. Reproduced with permission from American Chemical Society (modified from ref. [14]). (d) Schematic illustration of a BP/MoSe_2_ heterojunction device and response of three sensors as a function of NO_2_ gas concentration. Reproduced with permission from IOP Publishing [166]. (e) an all-carbon device fabricated on a flexible PEN substrate and magnified image of a 21-stage ring oscillator. Reproduced with permission from Springer Nature [171].

There are three dominant research directions for electronic applications of 2.5D materials. The first is next-generation complementary metal oxide semiconductor (CMOS) and memory technologies based on 2D materials integration. The most advanced transistor today is a fin-type field effect transistor (Fin-FET) [149–151], which can enhance gate-controllability and reduce power consumption. To further advance the technology node, the nanosheet gate-all-around (NS-GAA) structure is intensively investigated by reducing the thickness of fin width. Recently, the NS-GAA structure based on 2D heterostructures of MoS_2_/hBN/graphene was demonstrated [152], as shown in Figure 10(a). 2.5D heterostructures offer two main advantages here: one is their atomically thin (less than 1 nm) channel thickness, which cannot be reached with the conventional Si system; the other is that the surfaces of 2D materials are free of dangling bonds so that the hetero-interfaces are electrically inert. [153–156]. Therefore, the highest performance in terms of subthreshold swing (SS) and current on/off ratio has been realized, significantly improved over that of GAA structures containing conventional 3D channels made from Si, Ge, and other semiconductors. For memory technology, 2.5D heterostructures have been extensively investigated for next-generation non-volatile memory (NVM) devices, because very high electrical reliability is expected thanks to the electrically inert hetero-interface [157,158]. Recently, the memory operation mechanisms behind the round sweep-transfer curves have been revealed based on the systematic comparison of inherent floating gate voltage trajectories for three different 2D channels [159]. Moreover, ultrafast program/erase operation (~20 ns) has been demonstrated, as shown in Figure 10(b) [160,161]. Compared with the non-volatile floating-gate-type Si flash memory whose operation speed is the order of microseconds [162], 2.5D heterostructured NVMs are now knocking on the door of storage-class memory, with an operation speed close to that of working memory.

The second research direction is the ultra-low power consumption, which cannot be achieved by next-generation CMOS technology because of the thermodynamically limited steepness of the transfer characteristics in the subthreshold regime; that is, the SS is limited to be larger than 60 mVdec^−1^ at room temperature [161]. To overcome this limitation, two major concepts for steep-slope devices have been proposed: negative-capacitance (NC) FETs [163] and tunnel FETs (TFETs) [164]. TFETs are more feasible for 2.5D materials because the device design, which is based on the band-to-band tunnelling (BTBT), can be easily implemented by the characteristics of 2D materials – the shorter tunnelling distance owing to the vdW gap and the strong gate controllability owing to the atomically thin channel. All-2D-heterostructure TFETs (which may be called 2.5D-TFETs) produced by combining a type III *n*-MoS_2_/*p*^+^-MoS_2_ heterostructure with a hBN top gate insulator resulted in SS values lower than 60 mVdec^−1^ at room temperature, as shown in Figure 10(c) [14]. Further reductions in SS values and higher on-currents are possible when the entire hetero-interface is more rigorously controlled [165].

The third research direction is toward multifunctionality, to create, for example, sensors, transducers, and flexibility devices. As shown in Figure 10(d), using the 2.5D heterostructure of black phosphorus (BP) and MoSe_2_ layers, a highly sensitive gas sensor was demonstrated [166]. In general, a monolayer 2D channel is used as the sensing interface to provide an enhanced surface to volume ratio [167,168]. Here, the heterojunction demonstrates a considerably lower detection limit, ~25 ppb, and higher sensitivity toward nitrogen dioxide (NO_2_); this occurs because chemical adsorption can induce significant changes in band alignment and carrier transport behavior (Figure 10(d)). 2.5D heterostructures provide a new platform for sensing applications, where the different 2D materials that make up the heterostructure can actively and independently interact with target molecules and contribute to the sensing process. Moreover, flexibility is one of the advantages of 2.5D materials, some of which can accommodate strains exceeding 10%, giving an additional functionality to electronic applications [169,170]. Figure 10(e) displays an all-carbon device fabricated on a flexible polyethylene naphthalate (PEN) substrate [171]. This flexible device is fully transparent because the typical Au electrodes were replaced by carbon nanotube electrodes. Such transparency will drastically increase the range of applications. Although Figure 10(e) shows carbon nanotube device, the integration of the tubular materials (1D materials) with 2D materials [154] or inserting 1D materials within 2D nanospace [172] can open up a new paradigm for nanoscale material systems.

Finally, from the viewpoint of electronic applications of 2.5D materials, there is one issue which needs to be addressed, that is the smaller relative dielectric constant of multilayer hBN (~3) compared with that of SiO_2_ (3.9), which limits the usage of hBN as a gate insulator in ultrascaled CMOS devices [173]. Although enhancement of the capacitance by combining monolayer hBN and a high-*k* oxide is one of the possible solutions, a better option is likely to be exploring new 2D insulators, which would lead to a wide variety of applications without reducing the performance of 2.5D materials.

### 4.2. Energy and optoelectronic applications of 2.5D materials

2.5D materials are also being actively studied for application in energy conversion devices and optoelectronic devices. In TMDCs stacked with vdW heterojunctions, photoexcited electrons and holes are charge-separated via interfacial charge transfer. It is noted that monolayer TMDC shows a high light absorption efficiency of 5–10%, which is about 10 times higher than that of Si or GaAs [174]. Therefore, TMDC heterojunctions are expected to be applied in flexible photovoltaic cells and photodetectors. In addition, TMDC heterostructures are expected to be useful as catalysts and ion transfer membranes due to efficient electron transfer between the adjacent layers that are coupled by vdW interaction [175,176]. As well as this, TMDC and graphene can be applied to various energy conversion and light-emitting devices, where they offer new functions, such as plasmons and intercalation. They may also be used in optoelectronic devices in combination with 2D organic and inorganic hybrid lead halide perovskites (2D-PVSK). In this section, we review recent progress on the energy and optoelectronic applications of various 2.5D materials.

**Figure 11.**
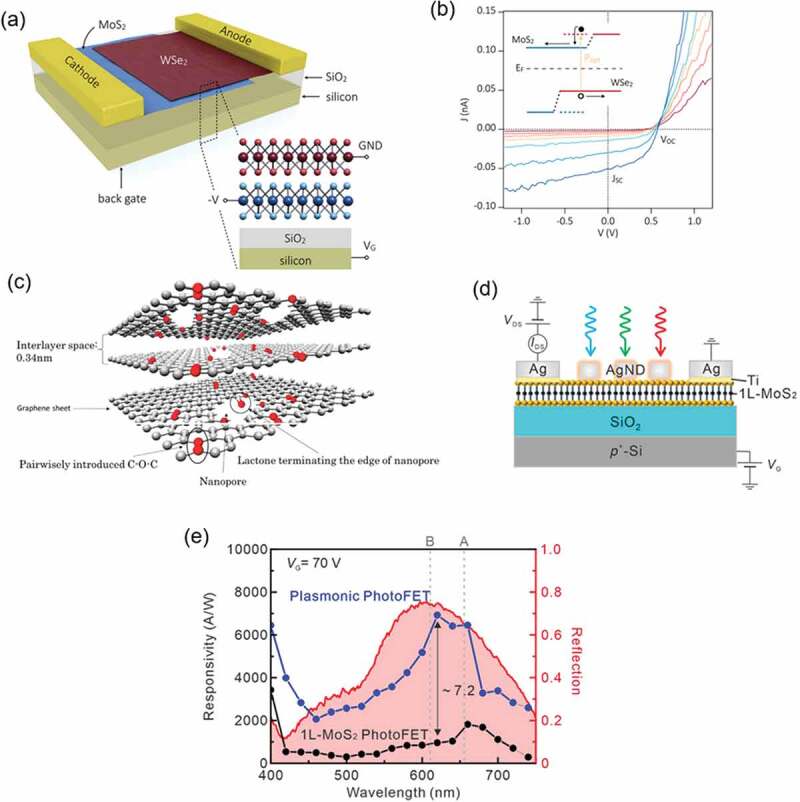
(a) Schematic illustration of the TMDC bilayer photovoltaic device. Reproduced with permission from American Chemical Society [177]. (b) *J*−*V* curves of the device shown in (a) measured under illumination of 180, 400, 670, 1100, 1800, 4000, and 6400 W/m^2^ (in the order of red to blue). Reproduced with permission from American Chemical Society [177]. (c) Schematic illustration of GLG. Reproduced with permission from Springer Nature [181]. (d) Schematic diagram of monolayer MoS_2_ plasmonic photoFets under bias and illumination with a gate voltage. Reproduced with permission from American Chemical Society [184]. (e) Photoresponsivity of the plasmonic photoFET and bare monolayer MoS_2_ photoFET as a function of illumination wavelength. The red curve shows reflection spectrum of the plasmonic nanostructures. Reproduced with permission from American Chemical Society [184].

In a photovoltaic device with a simple heterostructure of MoS_2_ and WS_2_, MoS_2_ acts as an electron acceptor and WS_2_ acts as an electron donor for charge separation. The unique point here is that the absorption spectrum is not a simple sum of the absorption spectra of the two materials – the existence of charge transfer excitons is predicted from theoretical calculations [174]. Furchi et al. constructed a MoS_2_/WSe_2_ photovoltaic system shown in Figure 11(a), and demonstrated the photovoltaic effect with an open circuit voltage of about 0.5 V (Figure 11(b)) [177]. Light is absorbed by both WSe_2_ and MoS_2_, and excitons are generated in both monolayers. Due to the spatial separation of the lowest energy electron and hole states, electronic excitation occurs across the heterojunction, followed by lateral diffusion of the generated carriers along the interface as shown in the inset of Figure 11(b) [177]. Interlayer recombination is supposed to occur during the carrier diffusion, reducing the photocurrent. Interestingly, Tan et al. theoretically proposed that charge recombination can be suppressed by inserting a 2–3 nm thick hBN insulating layer between the MoS_2_ and WSe_2_ and that the photoelectric conversion efficiency (PCE) of MoS_2_/hBN/WSe_2_ photovoltaic devices can be approximately doubled. They theoretically predicted that the PCE exceeding 23% could be obtained by increasing the TMDC thickness and optimizing the hBN thickness [178].

A MoS_2_/MoTe_2_ photovoltaic system using MoTe_2_ as an electron donor exhibited the photovoltaic effect with an open circuit voltage of 0.3 V by near-infrared light (800 nm) [179]. This can be explained by the charge separation mechanism based on the type-II band alignment, which is similar to the research by Furchi et al [177]. High-performance photovoltaic devices were also fabricated using a few-layer ReS_2_/monolayer WSe_2_ stack, achieving 1.5% PCE with a fill factor of 0.56 [180], an important step towards increasing the performance of flexible solar cells.

In a further example, BLG can be used to create a promising electrode material with high conductivity and transparency, by intercalating molecules and ions between the graphene layers. As already shown in Figure 5(d), intercalating MoCl_5_ molecules into twist-rich BLG significantly reduced the sheet resistance (83 Ω Ω^−1^), allowing to apply it to organic solar cells with a high PCE [79]. Graphene-like graphite (GLG, Figure 11(c), which has a 2D stacked, porous graphitic structure with chemically introduced C-O-C units, was applied as the anode in a Li-ion battery. The GLG electrode showed a large capacity of 608 mAh/g at the upper cell voltage limit of 2 V [181]. In contrast, MoS_2_ and MoSe_2_ nanosheets were applied as cathodes with Mg plate anodes for hybrid Mg-Li ion batteries [182]. The stacked structure of carbon-stabilized heat-expanded TMDC is expected to find applications in both the anode and cathode of carbon-free Li-ion batteries. Furthermore, MoS_2_ thin films are attracting interest as photoelectrodes for hydrogen evolution through water reduction because of their high light absorbance and high electrochemical activity [183].

Stacked structures of TMDC and metallic nanostructures that show localized surface plasmon resonances are another example of 2.5D materials that are expected to be used in light-energy conversion devices and optoelectronic devices. Lan et al. placed silver nanostructures on monolayer MoS_2_ to construct a gate-tunable plasmonic phototransistor (photoFET), as shown in Figure 11(d) [184]. The constructed plasmonic photoFET showed high photoresponsiveness, with a 7.2-fold increase (over that of the control non-plasmonic FET) at the plasmon resonance wavelength (Figure 11(e)). This is accounted for by the effective use of photons arising from the plasmon optical antenna effect and plasmon-induced photocarrier generation [184]. On the other hand, the electromagnetic field enhancement effect of the plasmons was also utilized for the light-emitting devices. Actually, the efficiency of the light-emitting devices was improved by exploiting the PL enhancement of MoS_2_ by plasmons [185,186].

**Figure 12.**
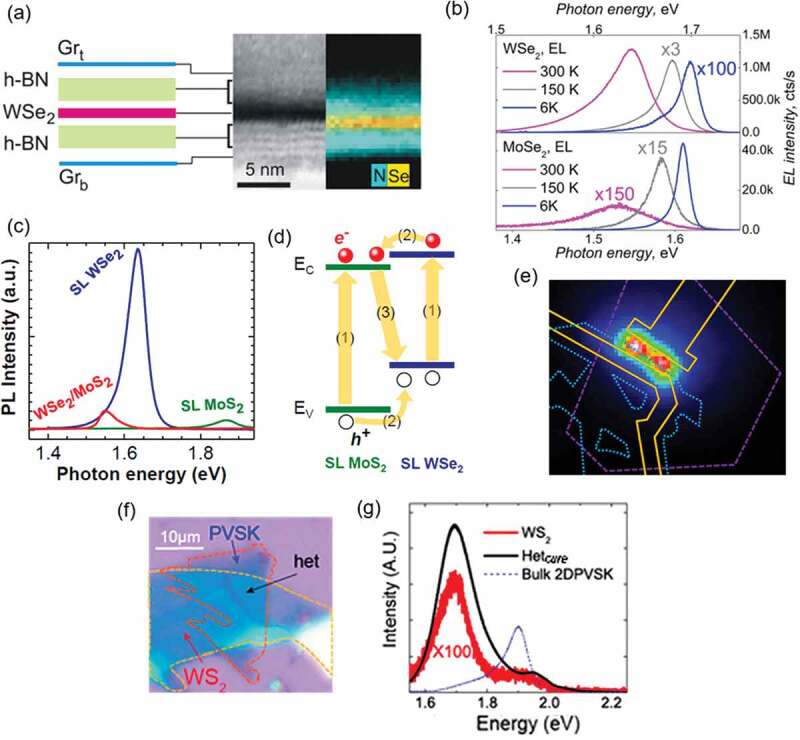
(a) Schematic of a light-emitting device sandwiching a single layer of WSe_2_ between hBN barrier layers with top and bottom transparent graphene electrodes. High-resolution TEM image and energy-dispersive X-ray analysis of a cross-section of the device. Reproduced with permission from American Chemical Society [188]. (b) Electroluminescence spectra taken at different temperatures for WSe_2_ and MoSe_2_ light-emitting devices with hBN tunnel barriers measured with applied bias of 2 V and 1.8 V, respectively. Reproduced with permission from American Chemical Society [188]. (c) PL spectra of single-layer WSe_2_, MoS_2_, and WSe_2_/MoS_2_ hetero-bilayer. Reproduced with permission from National Academy of Sciences [189]). (d) Energy diagram of WSe_2_/MoS_2_ hetero-bilayer under photoexcitation. Reproduced with permission from National Academy of Sciences [189]. (e) Electroluminescence from the WSe_2_/MoS_2_ hetero-bilayer light-emitting device. Reproduced with permission from American Chemical Society [139]. (f) Optical image of the bilayer-WS_2_/2D-PVSK (*n* = 4) heterostructure on a SiO_2_/Si substrate. Reproduced with permission from American Chemical Society [193]). (g) Photoluminescence spectra of the bare bilayer-WS_2_ (red line), the bare 2D-PVSK (*n* = 4) (blue), and bilayer-WS_2_/2D-PVSK heterostructure (black). The excitation wavelength is 514 nm. Reproduced with permission from American Chemical Society [193].

The 2.5D materials were applied not only to the photovoltaic devices but also to the light-emitting devices. In 2.5D materials science, it is possible to engineer band structures of materials by stacking various two-dimensional materials in a highly controlled manner. Even for light-emitting devices, stacking of mechanically exfoliated 2D materials ensures high quality in each of TMDC monolayers, so that high performance is generally obtained as a total device [187]. Withers et al. succeeded in demonstrating a light-emitting device with an external quantum efficiency (EQE) of 5% at room temperature by sandwiching a single layer WSe_2_ with h-BN tunnel barrier layers and top and bottom transparent graphene electrodes, (Figure 12(a)) [188]. Interestingly, the WSe_2_-based light-emitting device showed much higher EQE at room temperature than that of the MoSe_2_ device (Figure 12(b)). They showed diﬀerent temperature dependence, which was explained by the inverted sign of spin−orbit splitting of conduction band states in WSe_2_ and MoSe_2_ [188].

Hetero-bilayers of TMDC, such as WSe_2_/MoS_2_ system, has been well studied for the light-emitting device with analogous to photovoltaic devices. Single-layer WSe_2_ and MoS_2_ show PL spectra peaking at 1.64 eV and 1.87 eV, respectively, as shown in Figure 12(c). The WSe_2_/MoS_2_ hetero-bilayer gives the PL at 1.55 eV, which is related to the energy difference between the conduction band of MoS_2_ and the valence band of WSe_2_. Their band diagram is presented in Figure 12(d) [189]. By utilizing a hetero-bilayer of WSe_2_/MoS_2_ stack, Cheng et al. have successfully demonstrated the electroluminescence (EL) device (Figure 12(e)) [139].

Combinations of 2D-PVSK and TMDCs are other 2.5D materials that are also attracting attention for use in light-emitting devices. Erkılıç et al. succeeded in synthesizing the heterostack of 2D-PVSK and monolayer WS_2_ by all vapor phase deposition and observed the strong coupling between these different types of 2D materials from the modulation of their PL characteristics [190]. Optical transistors consisting of 2D-PVSK and WS_2_ have also been fabricated, and their efficient photovoltaic effects were investigated [191,192]. These materials also have some disadvantages – TMDCs have low quantum efficiencies, due to defect states and band-to-band transitions from direct to indirect, while 2D-PVSK is unstable in ambient atmosphere. Yang et al. reported that charge transfer states are formed when 2 L-WS_2_ is stacked on 2D-PVSK as shown in Figure 12(f); the PL intensity is increased by two orders of magnitude compared with that of bare WS_2_ and the stability is improved compared with that of bare 2D-PVSK (Figure 12(g)) [193]. Although this approach is still in its infancy, many studies have found that the characteristics of energy and optoelectronic devices can be improved by using 2.5D material systems.

## 5. Conclusions and future perspectives

**Figure 13.**
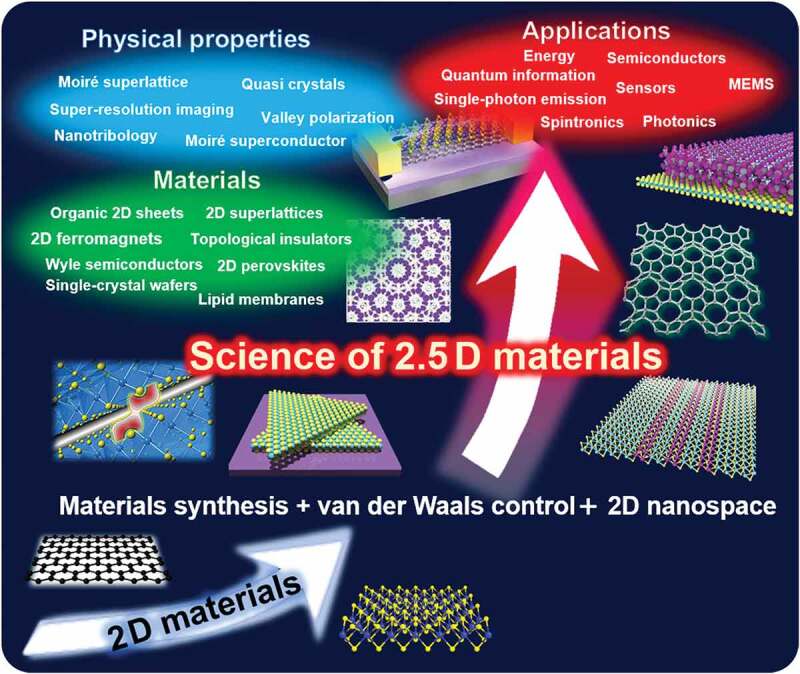
Future prospects of science of ‘2.5D materials.’

In this review, we explain our novel concept of 2.5D materials science by reviewing recent publications. This concept, which will further extend the current research on 2D materials, includes the synthesis of high-quality 2D materials and their wafers, sophisticated control of the stacking of various 2D materials, stacking of massive numbers of 2D materials with clean interfaces, finding and understanding novel phenomena, exploration of the science in interlayer 2D nanospace, integration of 2D materials with materials having different dimensions, and advanced research for applications to our life (3D). In addition to theoretical studies and calculations, other techniques, such as machine learning, deep learning, and materials informatics should be also incorporated to design and predict new 2.5D materials that exhibit outstanding properties and promising properties. As shown in Figure 13, there are many opportunities to explore new materials science, and potentially have a strong impact on our future life. This new concept also requires strong collaborations between researchers working in different fields. Through such collaborative research on the basis of ‘2.5D materials,’ we believe that the new scientific field will be established which lead to a new era of materials science.
